# Exploring HLA-C methylation patterns and nutritional status in Kichwa mothers and infants from Tena, Ecuador

**DOI:** 10.3389/fmed.2024.1356646

**Published:** 2024-08-27

**Authors:** Erick Velastegui, Isaac B. Falconí, Valeria I. Garcia, Gabriela Munizaga, Carmen Matias de la Cruz, Yaritza Segura, Kerly Alcivar, Luz Valencia, Edwin Vera, Mindy S. Muñoz, Wim Vanden Berghe, Sarah Lebeer, Andrea Orellana-Manzano

**Affiliations:** ^1^Escuela Politécnica Nacional, Departamento de Ciencias de los Alimentos y Biotecnología, Facultad de Ingeniería Química y Agroindustria, Quito, Ecuador; ^2^Escuela Superior Politécnica del Litoral, ESPOL, Laboratorio para investigaciones biomédicas, Facultad de Ciencias de la vida (FCV), ESPOL Polytechnic University, Guayaquil, Ecuador; ^3^Epigenetic Signaling Lab (PPES), Department of Biomedical Sciences, University of Antwerp, Antwerp, Belgium; ^4^Facultad de Medicina Clínica Alemana, Universidad del Desarrollo, Santiago, Chile; ^5^Department of Bioscience Engineering, University of Antwerp, Antwerp, Belgium

**Keywords:** HLA-C, epigenetic, nutrition, methylation, inheritance

## Abstract

Environment and lifestyle can affect the epigenome passed down from generation to generation. A mother’s nutrition can impact the methylation levels of her offspring’s epigenome, but it’s unclear which genes may be affected by malnutrition during gestation or early development. In this study, we examined the levels of methylated GC in the promoter region of HLA-C in mothers and infants from the Kichwa community in Ecuador. To do this, we analyzed saliva samples using bisulfite DNA sequencing. While we did not observe any significant differences in the mean methylation percentages in exon 1 of HLA-C between mothers and their infants after the first two years of lactation and life, respectively, we did find that infants tended to increase their methylation level during the first two years of life, while mothers tended to decrease it after the first two years of breastfeeding. When we compared methylation levels between mothers and infants using an ANOVA/posthoc Tukey test, we found that the average methylation for the entire population was less than 3% at T1 and T2. Although there was a tendency for infants to have higher methylation levels during their first two years of life and for mothers to have lower methylation levels after the first two years of breastfeeding, the mean values were not significantly different. However, we found a significant difference when we contrasted the data using a Kruskal-Wallis test at 0.05 for T1 AND T2 (*p*-value: 0.0148). Specifically, mothers had an average of X̅ = 2.06% and sons had X̅ = 1.57% at T2 (*p*-value: 0.7227), while the average for mothers was X̅ = 1.83% and for sons X̅ =1.77%. Finally, we identified three CpG motif nucleotide positions (32–33, 43–44, and 96–97) along the 122 bp analysis of HLA-C exon one, which was found to retain methylation patterns over time and is inherited from mother to offspring. Finally, our small pilot study did not reveal significant correlations between maternal and offspring nutritional status and DNA methylation levels of HLA-C exon one.

## 1 Introduction

“The study of all events leading to genetic unfolding and development” was the first concept of epigenetics coined by Conrad Hal Waddington more than 80 years ago. His well-known “Waddington landscape” has become a famous metaphor that visualizes the correlation between the changing nature of genes and the environment in which an individual develops and how these lead to changes in the phenotype of species (1). Interestingly, Waddington’s objective was to find the link between three branches of science, genetics, embryology, and evolution, even without knowing the molecular properties of DNA, as he only theorized that “environmental forces” must influence heredity. Over time, studies linking genome plasticity, the developmental conditions of an individual, and the phenotypic variation have resulted in a new research field, “Epigenetics.” Today, “Epigenetics” is defined as the study of heritable regulatory alterations of extranuclear, extracellular, psychological, or social origin that affect genetic activity without modifying the DNA nucleotide sequence of an individual, conditioning the level of expression of a specific region of the genome via chemical modifications of DNA/chromatin (methylation, acetylation,) with the ability to compact/relax DNA or change DNA binding affinity for regulatory pioneer transcription factors or ncRNA (microRNA, lncRNA) in promoter and/or structural regions of a gene (2–4).

Recent evidence suggests that epigenetic modifications may be equally or even more relevant than structural variations in DNA (5). One of the most studied epigenetic phenomena is DNA methylation, which has been linked as the primary cause or susceptibility factor in Alzheimer’s disease, psoriasis, Parkinson’s disease, diabetes, or cancer (4, 6). Methyl groups that covalently bind to the cytosine of DNA generate silencing patterns that can be inherited through cell mitosis and meiosis (7). Studies on the inheritance of methylation patterns by maternal lineage are fascinating, as during fertilization, the genome of paternal reproductive cells is largely demethylated before the first cell division, whereas most methylation patterns of the maternal genome are transferred by the action of methyltransferase (DNMT1) with the help of DNMT3A & DNMTT3B (8). Even so, during embryogenesis, a large part of the methylation of fetal DNA is erased by the action of ten-once translocases (TET), thymine-DNA glycosylase (TDG), and enzymes that modify histones, whereas methylation of imprinted genes or intergenerational transmitted patterns persists by the action of methyltransferase (DNMT1) (7, 9).

Differential gene expression of HLA-C has recently been observed between mothers and infants diagnosed with childhood mental and intellectual illnesses presumably caused by autophagy-induced oxidative stress or viral diseases during pregnancy (10). This highlights the importance of a healthy prenatal environment for the genetic stability of HLA-C and its expression levels in the offspring. Remarkably, HLA-C is considered the only gene of the major histocompatibility complex (MHC) expressed in the matrix between the fetus and the mother and actively participates in the cell cycle, triggering placental growth. As such, for adequate placental growth, there must be proper nutritional availability (11). In addition, HLA-C is the only member of the major histocompatibility complex (MHC) that acts as an immunoregulator during the migration of trophoblasts from the uterine lining to the endometrium, a crucial process in gestation since 30% of pregnancies in healthy people fail in this process (12).

It is estimated that the epigenetic memory of an individual conditions the phenotype of its offspring for evolutionary reasons that will be transmitted from one generation to another in a direct way and, in many cases, will be maintained for several generations (13). Undoubtedly, a mother’s lifestyle, nutrition, and stress will affect her epigenome, but there is still a lack of knowledge about the regions that remain methylated during the process of embryogenesis and how patterns or intergenerational DNA methylation are retained. Genes with many CpG islands susceptible to methylation and involved in a broad spectrum of pathologies are interesting candidates for further investigation to deepen our understanding of epigenetic inheritance. Here, we focused on the major histocompatibility complex class I, C (HLA-C) gene as an interesting research model to characterize epigenomic inheritance in mother–child pairs concerning their nutritional health status.

In this study, we present for the first time the DNA methylation regulation of exon 1 of the primary histocompatibility complex class I, C (HLA-C) in saliva samples of mothers and their infants during the first two years of child development concerning the nutritional status of members of the Kichwa culture in the Amazon region of Ecuador.

## 2 Materials and methods

### 2.1 Samples and ethical considerations

Saliva samples were obtained with the OG-500 Oragene kit for DNA and RNA from a total of 6 women from the Kichwa community and their six infants according to the guidelines approved by the Ethics Committee for Research on Human Subjects CEISH (2021-159E) of the Universidad San Francisco de Quito (USFQ) of Ecuador for the project “Longitudinal anthropological study on Food Patterns, Nutritional Status and Health Status of Breastfeeding/Lactating Mothers and their infants from Kichwa communities of Ecuador, using saliva and mucosal sequencing.” Individuals did not eat, drink, smoke, or brush their teeth 30 min before sampling as recommended by the manufacturer Oragene. A minimum of 2 mL of saliva was obtained. In the case of infants, saliva production was stimulated by gently massaging the outside of the cheeks, a protocol described and approved by CEISH (2021-159E).

### 2.2 Subjects and exclusion criteria

**Figure 1:**
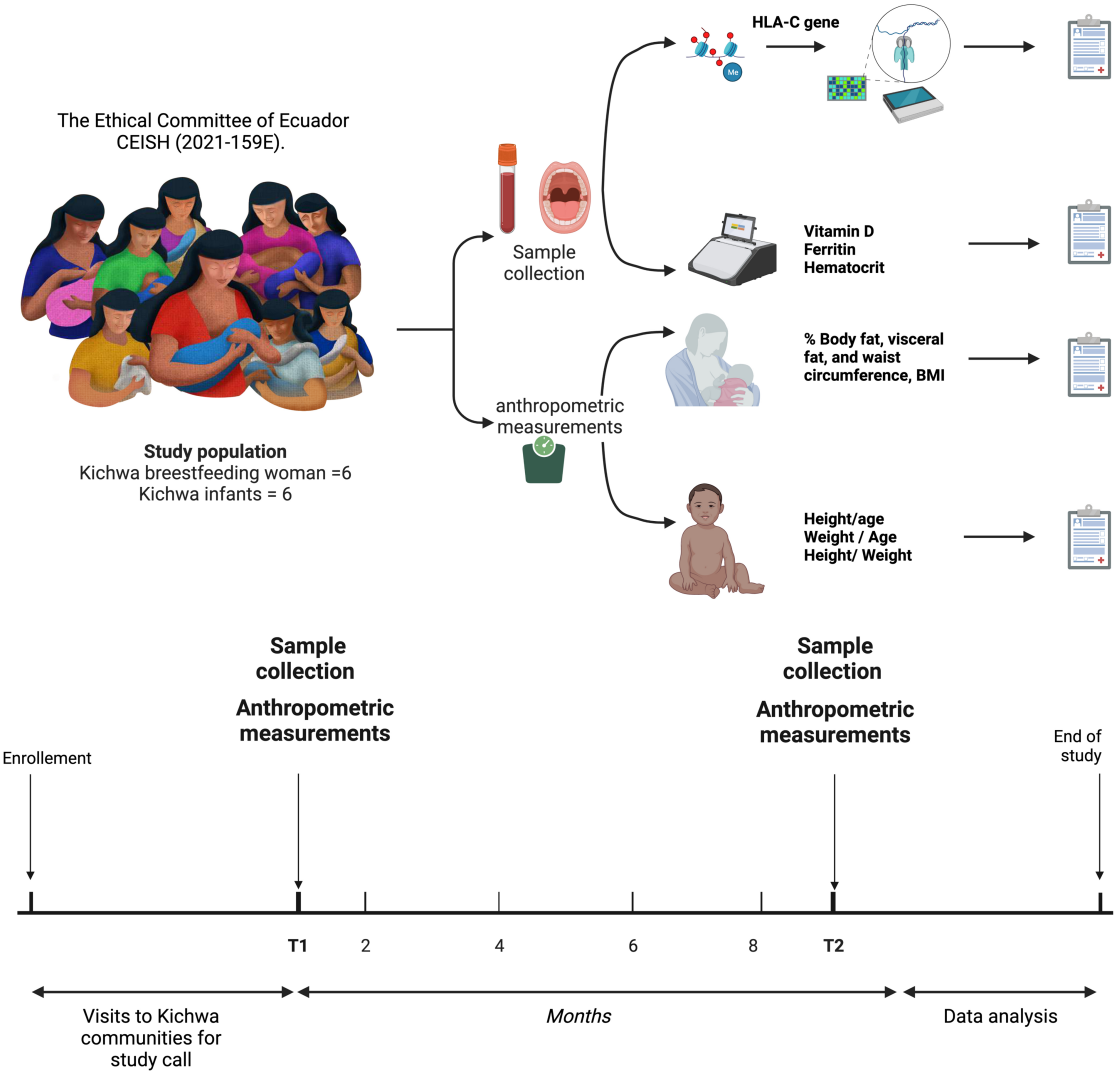
Sampling and sequencing procedure of HLA-C exon 1 in mothers and infants from the Napo province in the Ecuadorian Amazon.

The female participants were between 16 and 41 years of age, and the infants were between 1 and 6 months old in the first sampling, considered hereafter as T1, and 9 months later in the second sampling, considered hereafter as T2. All individuals were members of the Kichwa indigenous community for two or more generations, located in the Amazon of Ecuador in the province of Napo on the perimeter of the city of Tena. The age, weight, height, body mass index, fat percentage and diagnosis, muscle percentage, cardiovascular risk, ferritin, vitamin D, and hematocrit of the mothers, and the age, height, weight, ferritin, vitamin D, and hematocrit of the infants were recorded (Figure 1).

The parameters used to estimate obesity, overweight and hypertension are detailed in section 3 of the accompanying Supplementary material.

### 2.3 HLA-C sequencing

*Primer design*: The HLA-C sequence was obtained from the “Genome Browser” of the UCSC platform,^1^ for the design of the primers. Bisulfite PCR specific primers were designed on the EpiDesigner platform^2^ for amplification of (un)methylated bisulfite converted HLA-C exon 1 DNA (F: GGGGTTAGGGGGTTTTTTATATTTTTTTTAGA & R: CCTCCAAATAAACTCTCTCAACTACTACTCC). *Genomic DNA extraction*: DNA from mothers and infants was extracted with the PrepIT kit from Oragene kit OG-500 tubes with saliva samples. The DNA concentrations extracted from each piece were measured with Qbit, and the volume needed to obtain 300 ng of DNA from each sample was calculated for bisulfite conversion with the QIAGEN EpiTect Bisulfite Kit. PCR followed this with the QIAGEN PyroMark PCR Kit and electrophoresis to confirm the correct amplification of the bisulfite-converted HLA-C amplicon. The sequencing library was then prepared with 50 ng of DNA from each sample (4.66 μL on average) and made up to 9 μL with ultrapure water, and 1 μL of barcode from the Nanopore barcode 96 Kit Each sample was prepared and purified according to the manufacturer’s instructions. *Number of samples and technical replicas*: Twenty-four samples were sequenced in triplicate: 12 samples from T1 (6 mothers & 6 infants) and 12 from T2 (the same mothers and infants). *Sequencing depth*: 213,110 reads from three MinION r9.4.1 flow cells on a Nanopore Mk1c sequencer were generated. These reads were aligned and filtered, resulting in 162,744 (76.36%) high-quality alignments. The region coverage ranges from 10.13 to 42.68, estimated as the average number of reads that map against the reference using the Lander-Waterman equation (14).

### 2.4 Bioinformatic analysis of HLA-C with nanopore barcode 96 kit

The raw data underwent quality control using the FastQC tool (15). The adapters were removed by trimming them with the Porechop software (16) and filtering the sequences based on quality and size using the NanoFilt software (17). We set the minimum size to 44 bp, maximum length to 220 bp, and quality threshold to 14, with the options -l 44 --maxlength 220 and -q 14. We indexed the amplicon of the primer designed for sequencing with the Bismark-0.24.0 software (18) using the bismark_genome_preparation tool. The reads were mapped to our reference sequence using the minimap2 tool (19) with the nondirectional option to obtain outputs in BAM format. Methylation patterns were extracted with the bismark_methylation_extractor tool with the options bedGraph, buffer size 10G, cytosine_report. We generated methylation reports with bismark2report and bismark2summary tools. Finally, we visualized the mapped sequences using the IGV software (18).

### 2.5 Statistical analysis

We conducted statistical analyses on the methylation percentages for Epigenetics and methylation patterns using ANOVA and post-hoc Tukey tests, with adjustments made at 0.05 and 0.01 significance levels. Additionally, we utilized a non-parametric Kruskal-Wallis test at a 0.05 significance level. To determine the correlation between methylation and nutritional status, we used both Spearman’s and Pearson’s correlation coefficients. For the nutritional variables, we analyzed the qualitative variable using a chi-square test and the quantitative variable using ANOVA with Prism GraphPad 9 software.

The anthropometric and genetic raw data can be found in the attached Supplementary materials 1, 2.

## 3 Results

### 3.1 Nutritional status of Kichwa breastfeeding mothers and infants from Tena – Ecuador

Analyzing the results in Table 1, according to the anthropometric indicator BMI, we can observe that 66% of the Kichwa lactating mothers are overweight and 0% obese at T1. This nutritional status has been progressive because, at T2, there is already evidence of an increase from 0 to 16.7% of mothers with obesity. Consequently, according to the percentage of body fat, 100% of the women evaluated at T1 and T2 are diagnosed with obesity. Concerning cardiovascular risk, which is directly proportional to the increase in adipose tissue, medium risk rates of 33.3 and 16.7% are shown at T1 and T2, respectively. High cardiovascular risk predominates, identified in 50% of the population at both times. In addition, there is a significant difference over time for serum ferritin levels (*p*-value: 0.0043) and hematocrit (*p*-value: 0.0173), which show a higher median in T2, which may mean the presence of iron deficiency anemia in lactating women.

**Table 1:** Nutritional characteristics of Kichwa lactating women with Kruskall-Wallis.

	T1		T2		*p* value
	*N* = 6		*N* = 6		
BMI diagnosis, *N* (%)					
Normoweight	2	33.3	2	33.3	0.6276
overweight	4	66.7	3	33.3	
Obesity	0	0	1	16.7	
Fat percentage, *N* (%)					
Average	0	0	0	0	0
Obesity	6	100	6	100	
Cardiovascular risk, *N* (%)					
Low	1	16.7	2	33.3	0.7277
Moderate	2	33.3	1	16.7	
High	3	50	3	50	
**Ferritin, median (IQR)**	10.57	(10–10.86)	37.90	(10.16–68.55)	0.0043*
**Vitamin D, median (IQR)**	60.68	(35.14–64.29)	27.76	(20.19–47.07)	0.132
**Hematocrit, median (IQR)**	47	(43.25–48.25)	39	(36.75–41.25)	0.0173*
**Muscle %, median (IQR)**	27.70	(25.80–30.25)	27.80	(25.40–28.85)	0.7922
**H/w %, median (IQR)**	0.88	(0.82–0.92)	0.84	(0.8–0.95)	0.2987

^*^There is a significant difference.

On the other hand, most of the Kichwa infants at T1 showed an adequate BMI for their age, except for 16.7% who were emaciated; however, at T2, there was a radical change with the results obtained previously, since 33.3% showed an adequate weight, while 66.7% of the population showed high rates of risk of overweight. In addition, Vitamin D deficiency was observed because serum levels of Vit D and ferritin are significantly different over time (Table 2).

**Table 2:** Nutritional characteristics of Kichwa infants with Kruskall-Wallis.

	T1		T2		*p* value
	*N* = 6		*N* = 6		
**Age, weeks**	10.25	(2.025–13.08)	117.3	(98.40–119.9)	0.0022*
Weight/age interpretation					
Adequate	6	100	6	100	
Overwieight risk	0	0	0	0	0
Emaciated	0	0	0	0	
Height/age interpretation					
Adequate	5	83.3	6.0	100.0	0.4
High	1	16.7	0.0	0.0	
BMI/age Interpretation					
Adequate	5	83.3	2.0	33.3	0.0
Overwieight risk	0	0.0	4.0	66.7	
Emaciated	1	16.7	0.0	0.0	
**Ferritine, median (IQR)**	50.76	(30.05–90.66)	14.47	(10–42.87)	0.0476*
**Vitamin D, median (IQR)**	70.00	(67.57–70)	24.09	(15.64-45.78)	0.0152*
**Hematocrit, median (IQR)**	34.50	(32.75-39.25)	34.50	(33.75-38.25)	0.80

^*^There is a significant difference.

### 3.2 Epigenetics and methylation patterns of mothers and infants

**Figure 2:**
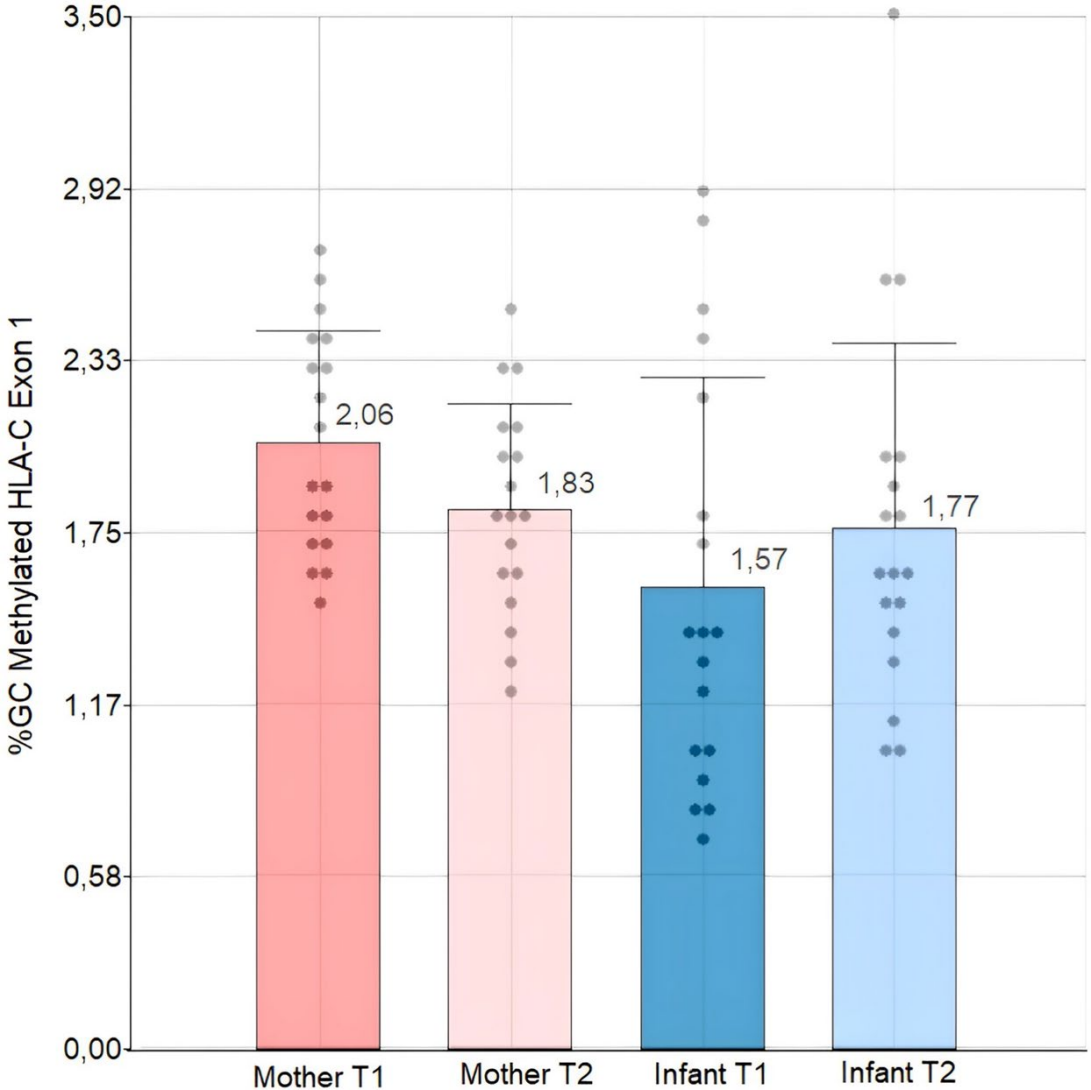
Bar and dot plot with standard deviation and dispersion data of HLA-C Exon 1 methylation levels in Kichwa mothers and infants at T1 and T2. T1: time 1 T2: time 2. We performed a Kruskal-Walli’s test with a 0.05 significance level.

Upon analyzing the relative HLA-C exon1 DNA methylation levels (%) of individuals at T1 and T2, we discovered that the average mean methylation of the Kichwa population is less than 3%. Upon analyzing the data of the mean percentage of % GC methylation, we found no statistically significant differences between T1 and T2 for both mothers and infants. This was done through an analysis of variance and a post-hoc Tukey test, adjusted to a 0.05 and 0.01 level of significance. However, we noticed a tendency for infants to increase their methylation level in the first two years of life, while mothers tended to decrease it after the first two years of breastfeeding. Moreover, the methylation percentages of mothers and infants were significantly different (*p*-value: 0.0332) according to our Kruskal-Wallis test at a 0.05 significance level (Figure 2).

**Figure 3:**
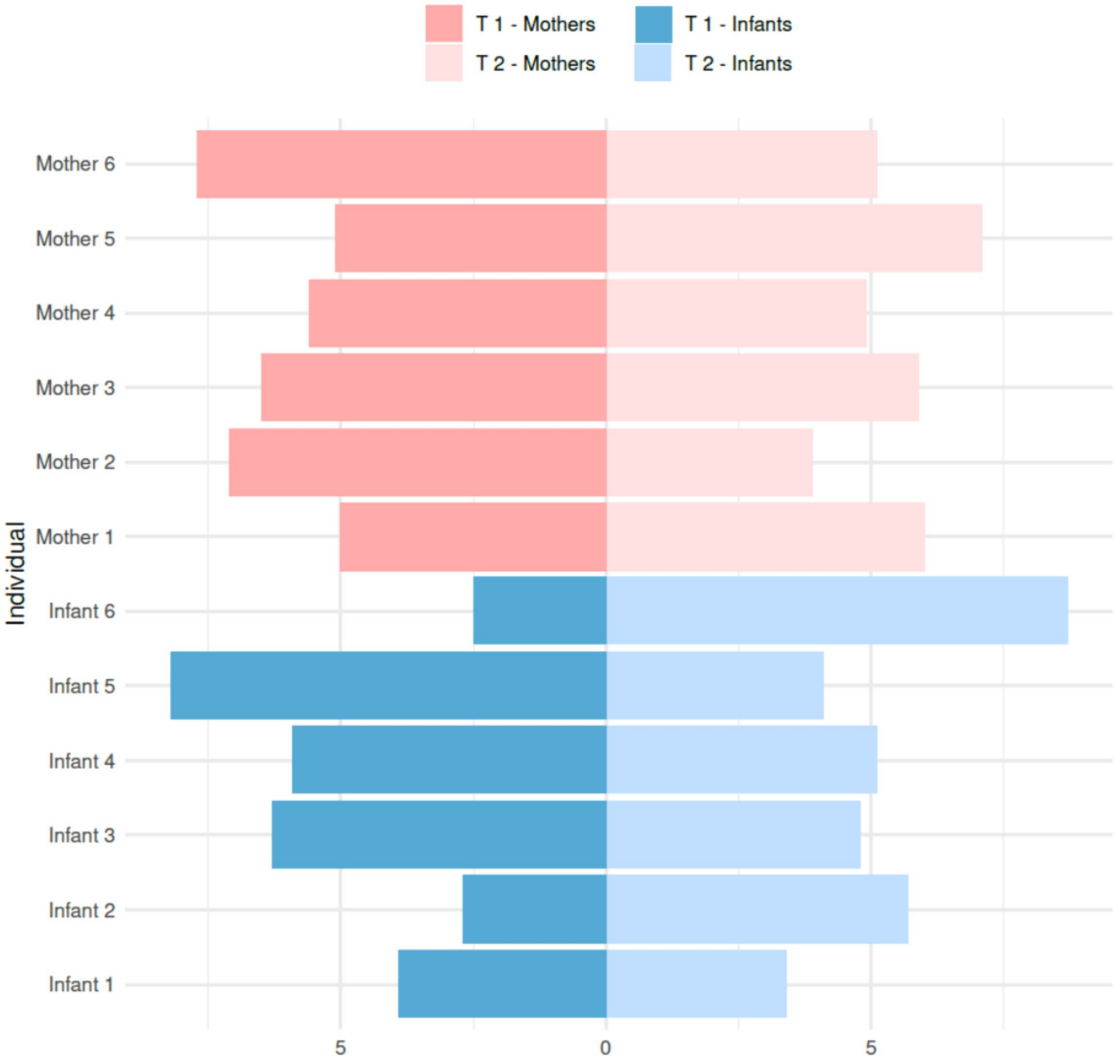
Bilateral plot of absolute methylation levels (%) in HLA-C exon 1 of individual Kichwa mothers and infants at T1 and T2.

At the individual level, we observed some changes when comparing the mean methylation levels of HLA-C exon 1 in each of the 12 individuals between T1 and T2. Infant 2 showed an increase from 0.90 to 1.90%, and infant 6 had the highest increase in methylation, going from 0.83 to 2.90%. Conversely, Infant 5 significantly decreased methylation, falling from 2.73 to 1.37%. As for the mothers, mother one and mother five both increased their methylation levels from 1.67 to 2.00% and 1.7 to 2.37%, respectively (Figure 3). However, mothers 2 and 6 decreased their methylation levels from 2.37 to 1.30% and 2.57 to 1.70%, respectively.

**Figure 4:**
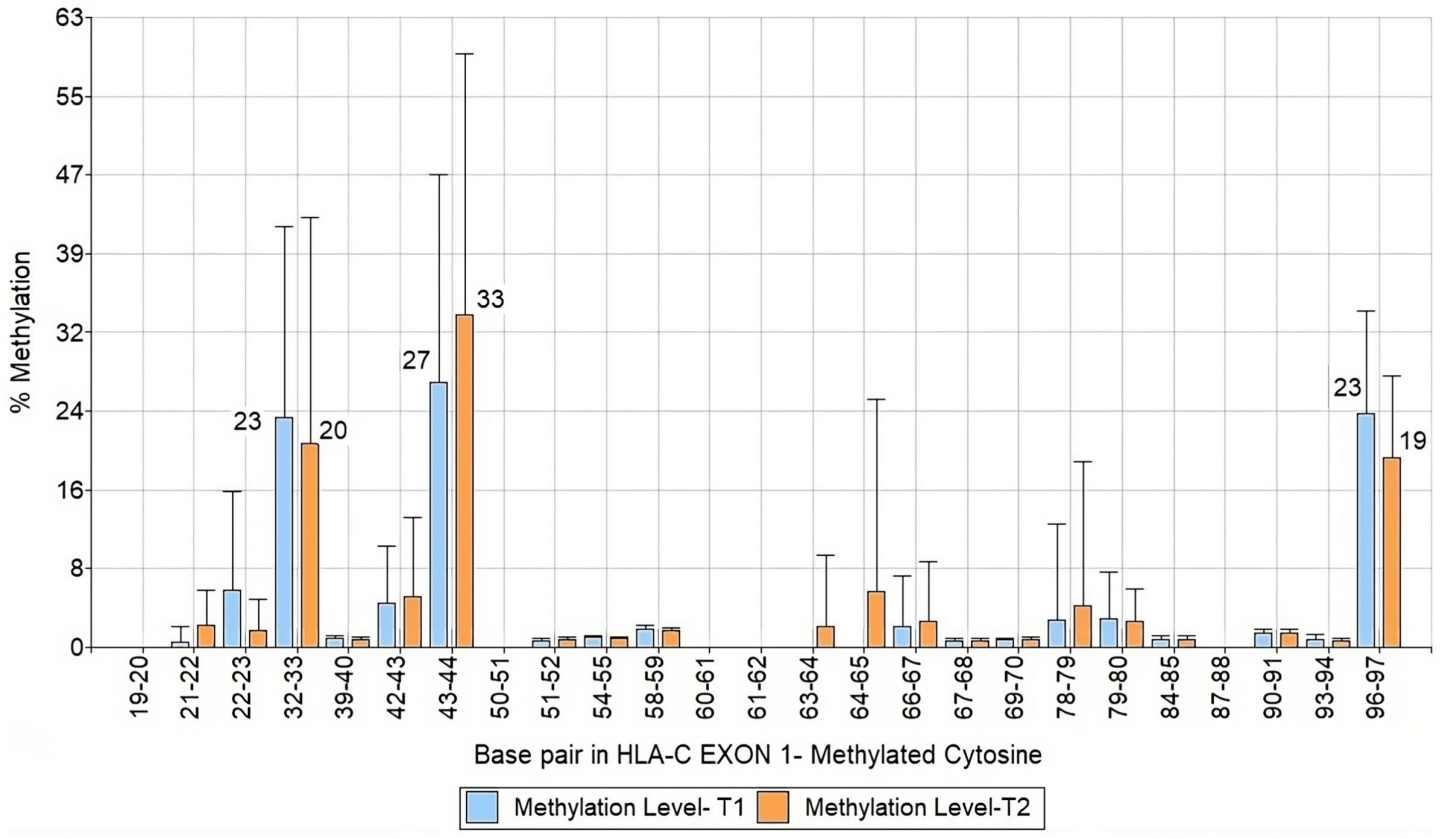
Bar chart of HLA-C exon 1 methylation patterns of infants and mothers averaged T1 and T2.

**Figure 5:**
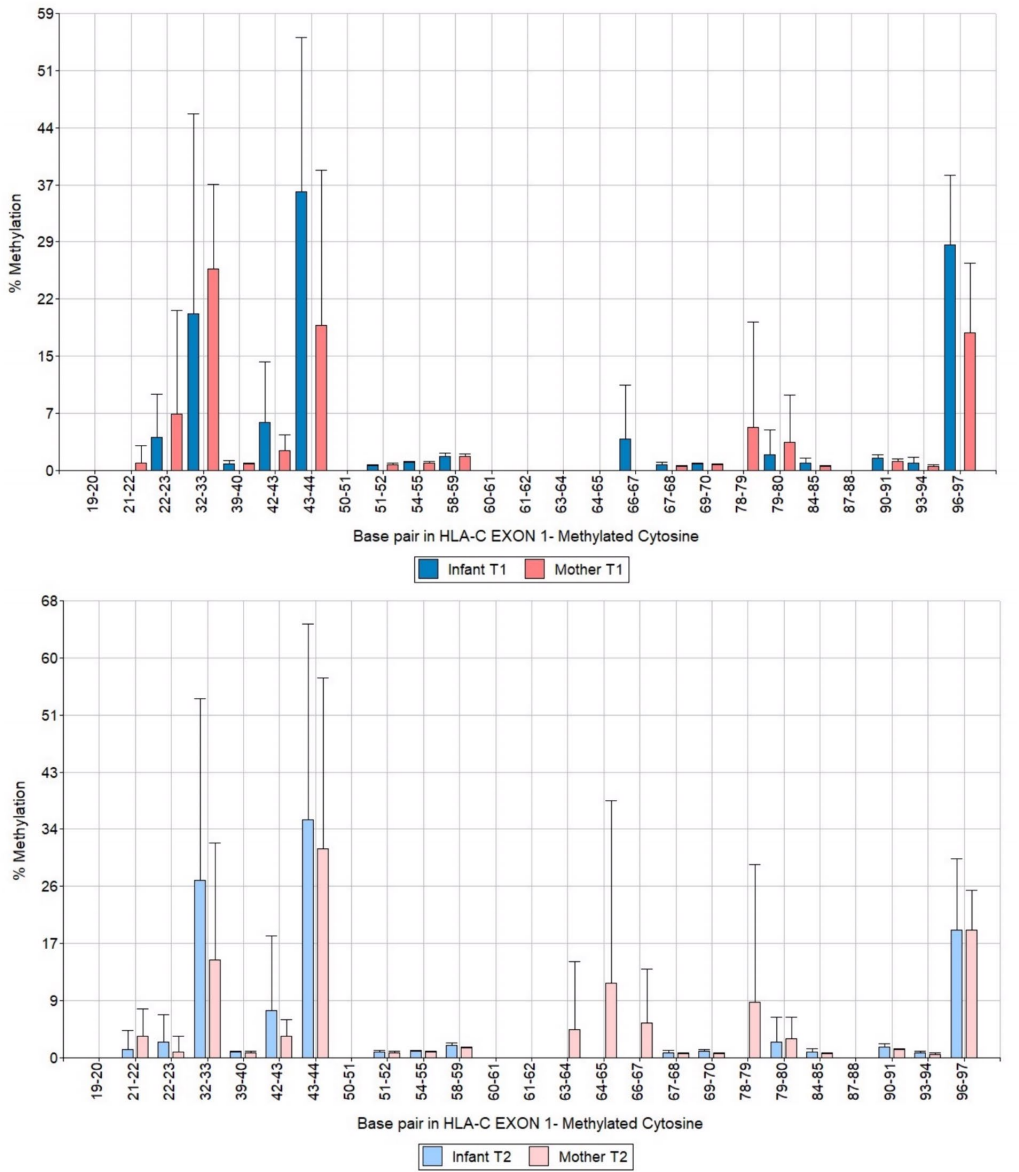
Bar chart of HLA-C exon 1 methylation patterns of infants and mothers contrasting T1 and T2 times.

We analyzed a 122 bp segment of exon 1 of HLA-C covering 25 CpG sites. Among them, three were hypermethylated, specifically CpG motifs 32–33, 43–44, and 96–97 (Figure 4). When we compared the data of mothers and infants in T1 and T2, we observed that the methylation levels remained stable over time and were inherited through the maternal line (Figure 5). These findings shed light on the methylation patterns of HLA-C and provide insight into the potential role of maternal inheritance in this process.

### 3.3 Correlation of methylation and nutritional status of mothers and infants Kichwa

During the study, it was observed that one out of 6 Kichwa mothers had a moderate cardiovascular risk at T1, which decreased again by T2, corresponding with a decreased methylation percentage of 2% (*p*-value: 0.0001), Table 3. Meanwhile, the BMI showed a significant upward trend, with four mothers being overweight at T1 and only one mother being obese at T2, with a methylation percentage of 1.8% (*p*-value: 0.0065).

**Table 3:** Nutritional characteristics of Kichwa mothers and infants with their % HLA-C methylation level.

		T1		T2		*p* value
		*N*	Media (IQR)	*N*	Media (IQR)	
Kichwa Mothers	Cardiovascular risk, *N* (%)					
	Low	1	1.7 (1.5–1.8)	2	2 (1.8–2.1)	ns
	Moderate	2	2.3 (2.123–2.425)	1	1.3 (1.2–1.4)	0.0001*
	High	3	1.9 (1.650–2.7)	3	1.8 (1.6–2.5)	ns
	BMI Diagnosis, *N* (%)					
	Normoweight	2	1.7 (1.5–1.8)	2	2 (1.875–2.1)	0.0078*
	Overweight	4	2.3 (1.950–2.475)	3	1.8 (1.350–2.3)	0.0065*
	Obesity	0	NS	1	1.6 (1.6–1.7)	ns
	Height/age interpretation					
Kichwa infants	Adequate	5	1.3 (1.2–1.4)	6.0	1.8 (1.5–2)	ns
	High	1	1.4 (0.9–2.4)	0.0	NS	
	Weight/age interpretation					
	Adequate	6	1.4 (1.0–1.96)	6.0	1.7 (1.608–1.850)	ns
	Overwieight risk	0	NS	0.0	NS	
	Emaciated	0	NS	0.0	NS	

^*^There is a significant difference. NS, no subject; ns, no significate.

Three of six Kichwa infants maintained an adequate weight at T1, with a methylation percentage of 1.55 (*p*-value <0.0001). The underweight infant at T1 became average weight at T2 with a methylation percentage of 1.8.

**Figure 6:**
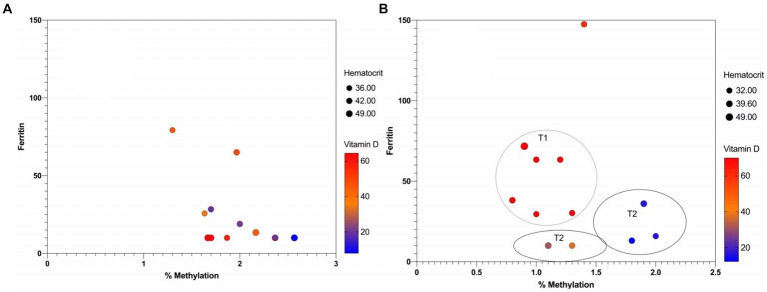
Multicomponent plot of nutritional variables and methylation percentage: **(A)** Mothers and **(B)** infants.

Furthermore, we analyzed whether there is any relationship or correlation between the percentage of methylation and three specific variables: ferritin, vitamin D and hematocrit. However, no statistically significant correlation could be detected between mean percentage methylation and ferritin, vitamin D and hematocrit in mothers over time (Figure 6A).

In contrast, in infants, an inverse correlation could be observed between the percentage of methylation and vitamin D over time, with a p-value = 0.0039 (Figure 6A). At T1, there is a low percentage of methylation with vitamin D values close to 60 mg/DL, while at T2, there is hypermethylation with low vitamin D values. This correlation between high levels of methylation and low levels of vitamin D expression in infants is one of the first approximations of epigenetic inheritance in HLA-C. Still, it must be considered that it was performed in a population of only six infants.

## 4 Discussion

We report on the first anthropometric characterization of a Kichwa mothers’ population in Tena, which analyzed Body Mass Index (BMI), body fat percentage, and cardiovascular risk, besides measurement of (micro)nutritional parameters such as ferritin, vitamin D, and hematocrit levels. A higher rate of overweight and obese mothers was also observed, which may be at increased risk of developing cardiovascular disease in the long term. Additionally, deficiencies of essential nutrients may further predispose them to specific pathologies such as iron deficiency anemia or metabolic disorders (20, 21).

Vitamin D deficiency is associated with increased autoimmunity and susceptibility to infections (22); it is important to mention that the relevance of HLA-C in autoimmune diseases has been described in detail in previous studies (23). Vitamin D has been shown to promote a tolerogenic immune response and may have a protective effect in autoimmune diseases (24). Vitamin D has been found to regulate the expression of HLA-C and other MHC class I genes, suggesting that vitamin D may influence the immune response by modulating antigen presentation to T cells (25). In addition, vitamin D has been shown to positively regulate genes related to the immune system while negatively regulating genes involved in cellular metabolism (25). However, little research reports the link between methylation levels and vitamin D in infants, even though vitamin D is essential for skeletal and immune system development in the first years of life. Therefore, in this study, we propose a possible correlation between HLA-C methylation and vitamin D deficiency; however, it is essential to evaluate in the future the whole set of MHC major histocompatibility complex genes or an analysis of the entire epigenome in mothers and infants to understand better this phenomenon of epigenetic inheritance and vitamin D deficiency. Again, it is important to mention that these results are estimates because of the number of the population studied.

During breastfeeding, the infants receive significant nutrients from the mother’s milk. Therefore, the mother must maintain a balanced diet to prevent any nutritional or energetic deficiencies in their child. Besides, it is equally important that this diet is diverse; otherwise, the mother may be at risk of developing obesity accompanied by nutritional deficiencies (26). Up to 25% of pregnant women may face metabolic diseases, and in some populations like India, up to 33% of women experience maternal diabetes due to suboptimal diet (27). This scenario may be similar to the situation in Eastern Ecuador, the region with the highest malnutrition rate in the country. In this study, we found that the anthropometric factors of mothers indicate a tendency to acquire metabolic diseases and pathologies that their offspring could inherit and also precondition their quality of life and disease risk in the long term (28, 29).

It is important to note that lactating women who suffer from iron deficiency anemia may experience a depletion of their essential iron reserves. This condition can be attributed to a lack of folic acid supplementation from gestation to lactation (30). It’s important to note that a mother’s insufficient micronutrient consumption can directly affect her baby’s nutritional status through breast milk. This can result in deficient levels of ferritin and vitamin D in infants, which puts them at risk for conditions like low weight, low height, anemia, rickets, and other health problems (31).

The proposed mechanism for estimating the correlation between HLA-C gene methylation and the immune system is that DNA methylation patterns undergo significant changes in the first months of life, which could be associated with environmental factors and the developmental process during early life such as vitamin D level (32). However, the molecular signaling mechanisms between vitamin D deficiency and methylation are not yet described. It is possible to determine the methylation of the immune system in saliva because DNA particles can be found in saliva (33). We use saliva containing salivary gland epithelial cells (SGEC), which can be used to study DNA methylation patterns (33). However, it is important to note that methylation patterns in saliva may not necessarily reflect methylation patterns in other tissues or organs, but it has the advantage that it is easily obtained and preserved with the appropriate kit, especially when sampling in the pediatric population.

Although no significant differences in mean methylation changes of HLA-C exon1 were detected, we identified a tendency for infants to increase their methylation levels with age. This trend is supported by evidence from epigenetic drift, which suggests that DNA methylation tends to increase over time in correlation with age and various lifestyles (34). In a study of infants, researchers also found increased DNA methylation over time (35). The research suggests that DNA methylation patterns undergo significant changes in the first few months of life. This could be associated with environmental factors and the developmental process during the early stage of life. The study also indicates a previously unreported transgenerational stability of HLA-C exon 1 methylation level from mother to child.

Finally, it should be mentioned that small variations in DNA methylation can only be relevant if we start from enough reads. This is to achieve significant accuracy in detecting a minimum percentage of changes in DNA methylation, and the nanopore sequencing used in this study allowed us to obtain several reference sequences for the calculation of coverage at a cost per sample considerably lower than other types of sequencing. The minimal difference in DNA methylation with biological relevance is still debated in the epigenetics community, but the future of identifying methylation patterns with nanopore technology is promising.

It is important to note that mothers can also influence the changes in infant DNA methylation. Maternal diet, including daily feeding and supplementation, can impact infant DNA methylation, especially in genes related to metabolism, development, appetite regulation, and maintenance of DNA methylation (36). Changes in DNA methylation patterns are associated with metabolic conditions such as obesity, insulin resistance, diabetes, and other metabolism-related diseases (37). Future studies need to focus on the nutritional impact and methylation levels of the entire epigenome, considering the diet of different populations and the intestinal microbiome, which is transcendental in correctly assimilating nutrients. It is still unclear how the epigenome is inherited. However, studies focusing on genes with high immune and pathological relevance, such as HLA-C, are highly relevant to better understanding the epigenetic implications of healthy motherhood. Our study is the first anthropometric report that characterized inherited methylation patterns in HLA-C exon1 in mother–child pairs in the Kichwa population associated with various nutritional parameters. Future epigenome-wide studies in larger cohorts are warranted to study the possible trans-intergenerational epigenetic impact of breastfeeding motherhood on health status during childhood and long-term trajectories upon growing up and aging.

## 5 Conclusion

Our team has conducted an innovative study that analyzes the physical attributes of a cohort of Kichwa mothers residing in Tena. The study delved into various parameters, including Body Mass Index (BMI), body fat percentage, cardiovascular risk, and levels of vital nutrients like ferritin, vitamin D, and hematocrit. The study discovered that a higher percentage of mothers in the group were overweight or obese, which elevates their risk of developing long-term cardiovascular diseases. Additionally, the study revealed that deficiencies in essential nutrients, particularly vitamin D, could make them more prone to autoimmune disorders. Furthermore, a possible correlation between HLA-C gene methylation and vitamin D deficiency was identified, emphasizing the necessity for comprehensive analyses of the entire epigenome. The study highlights the crucial role of maternal nutrition since deficiencies, especially in iron and folic acid, can impact maternal health and the nutritional status of infants through breastfeeding. The study also provides a new perspective on the relationship between HLA-C exon1 methylation and immune system development in infants. Although the limitations of studying methylation patterns in saliva are recognized, the research suggests the transgenerational stability of HLA-C exon1 methylation from mother to child.

Additionally, the research revealed that nanopore sequencing presents a cost-effective and precise way of detecting alterations in DNA methylation. The study highlights the crucial role of maternal influence on the methylation of infant DNA, mainly through diet, underscoring the call for more investigation into the nutritional effects and epigenetic consequences of healthy motherhood. Ultimately, this groundbreaking study establishes a basis for comprehending the intricate interplay of genetics, nutrition, and epigenetics in the Kichwa community, as well as paving the way for more extensive and comprehensive studies on the transgenerational impact of breastfeeding on health outcomes.

## Data availability statement

All data used for analysis are in the supplementary material. No personal data of the population was included due to the privacy of the indigenous community studied.

## Ethics statement

The study was conducted under the acceptance of the Human Research Committee CEISH (2021-159E) of the Universidad San Francisco de Quito (USFQ) of Ecuador for the project “Anthropological longitudinal study, dietary patterns, nutritional status and health status of nursing mothers and their infants from Kichwa communities, using saliva and mucosa sequencing”. The studies were conducted in accordance with the local legislation and institutional requirements. Written informed consent for participation in this study was provided by the participants’ legal guardians/next of kin.

## Author contributions

ErV: Conceptualization, Investigation, Writing – original draft. IF: Data curation, Investigation, Methodology, Software, Writing – original draft. VG: Formal analysis, Investigation, Methodology, Writing – review & editing. GM: Data curation, Methodology, Validation, Writing – review & editing. CM: Formal analysis, Investigation, Methodology, Writing – review & editing. YS: Data curation, Formal analysis, Investigation, Methodology, Writing – review & editing. KA: Investigation, Methodology, Writing – review & editing. LV: Writing – review & editing, supervision, coceptualization. EdV: Supervision, Validation, Visualization, Writing – review & editing. MM: Data curation, Software, Supervision, Validation, Visualization, Writing – review & editing. WV: Data curation, Funding acquisition, Resources, Supervision, Validation, Visualization, Writing – review & editing. SL: Resources, Software, Supervision, Validation, Visualization, Writing – review & editing. AO-M: Funding acquisition, Investigation, Project administration, Resources, Supervision, Writing – review & editing.

## Footnotes

^1^ https://genome.ucsc.edu/cgi-bin/hgGateway

^2^ https://www.epidesigner.com/
