# Supplementary material for: Exploring HLA-C methylation patterns and nutritional status in Kichwa mothers and infants from Tena, Ecuador
Source: Front Med (Lausanne). 2024 Aug 27;11:1356646. doi: 10.3389/fmed.2024.1356646 (PMC11385616; doi:10.3389/fmed.2024.1356646)
Supplement: Supplementary file 1 [file Data_Sheet_1.PDF]

Supplementary Material

Description: Raw data taken at T1 and T2 with percentages of GC methylated in exon 1 of HLA-C from (1) Kichwa mothers and (2) infants.

Section 1: MOTHERS

Sampling Year	Mothers	**Individual**	**Sample code**	**% CpG AVARAGE**	**%CpG methylation 1**	**%CpG methylation 2**	**%CpG methylation 3**	**Length HLA-C (bp)**	**IMC DIAGNOSIS**	**DIAGNOSIS % FAT**	**% MUSCLE**	**CARDIOVASCULAR RISK**	**Ferritin g/ml**	**Vitamine D**	**Hematocrit**
2019	Mother	**Mother 1**	**M22**	1.73	1.8	1.7	1.5	240	NORMAL	OBESITY	27.9	MODERATE	10	64.23	48
2019	Mother	**Mother 2**	**M23**	2.3	2.3	2.3	2.5	241	OVERWEIGHT	OBESITY	30.7	MODERATE	10	62.85	45
2019	Mother	**Mother 3**	**M18**	2.08	1.9	2.4	2.2	239	OVERWEIGHT	OBESITY	25.5	MODERATE	13,43	44.19	48
2019	Mother	**Mother 4**	**M30**	1.74	1.6	1.9	2.1	244	OVERWEIGHT	OBESITY	30.1	HIGHT	10	58.51	38
2019	Mother	**Mother 5**	**M29**	1.69	1.6	1.8	1.7	243	NORMAL	OBESITY	25.6	HIGHT	10	64.48	46
2019	Mother	**Mother 6**	**M32**	2.48	2.4	2.6	2.7	239	OVERWEIGHT	OBESITY	27.5	HIGHT	10	8	49
2021	Mother	**Mother 1**	**M60**	1.96	2	1.9	2.1	244	NORMAL	OBESITY	28.8	LOW	18.93	21.1	41
2021	Mother	**Mother 2**	**M68**	1.29	1.2	1.4	1.3	245	OVERWEIGHT	OBESITY	27.8	MODERATE	79.25	46.44	36
2021	Mother	**Mother 3**	**M53**	1.85	1.8	2	2.1	242	NORMAL	OBESITY	28.9	LOW	64.98	48.95	42
2021	Mother	**Mother 4**	**M63**	1.63	1.6	1.7	1.6	249	OBESITY	OBESITY	24.1	HIGHT	25.71	34.41	37
2021	Mother	**Mother 5**	**M52**	2.35	2.3	2.5	2.3	243	OVERWEIGHT	PREGNANCY	PREGNANCY	HIGHT	10.16	20.59	38
2021	Mother	**Mother 6**	**M38**	1.66	1.5	1.8	1.8	241	OVERWEIGHT	OBESITY	26.7	HIGHT	28.38	19.4	40

Section 2: INFANTS

Sampling Year	Mothers/ IFANTS	**Individual**	**Sample code**	**% CpG AVARAGE**	**%CpG methylation 1**	**%CpG methylation 2**	**%CpG methylation 3**	**Length HLA-C (bp)**	**WEIGHT INTERPRETATION**	**INTERPRETATION OF THE SIZE**	**Ferritin ng/ml**	**Vitamin D**	**Hematocrit**
2019	Infante	**Infant 1**	**I22**	1.25	1.2	1.4	1.3	242	ADEQUATE WEIGHT	HIGHT	63.36	70	34
2019	Infante	**Infant 2**	**I23**	0.86	0.9	0.8	1	241	ADEQUATE WEIGHT	ADEQUATE	147.48	60.26	35
2019	Infante	**Infant 3**	**I18**	2.27	2.4	2.2	1.7	243	ADEQUATE WEIGHT	ADEQUATE	30.22	70	33
2019	Infante	**Infant 4**	**I30**	1.44	1.4	1.4	1.8	244	ADEQUATE WEIGHT	ADEQUATE	71.72	70	49
2019	Infante	**Infante5**	**I29**	2.78	2.8	2.9	2.5	246	ADEQUATE WEIGHT	ADEQUATE	38.16	70	36
2019	Infante	**Infant 6**	**I32**	0.81	0.8	0.7	1	247	ADEQUATE WEIGHT	ADEQUATE	29.54	70	72
2021	Infante	**Infant 1**	**I60**	1.07	1	1.1	1.3	244	ADEQUATE WEIGHT	LOW	63.41	68.38	43.17
2021	Infante	**Infant 2**	**I68**	1.92	2	1.8	1.9	242	ADEQUATE WEIGHT	ADEQUATE	10	31.4	58
2021	Infante	**Infant 3**	**I53**	1.42	1.4	1.6	1.8	240	ADEQUATE WEIGHT	ADEQUATE	10	37.71	34
2021	Infante	**Infant 4**	**I63**	1.68	1.6	2	1.5	244	ADEQUATE WEIGHT	ADEQUATE	15.87	16.75	33
2021	Infante	**Infant5**	**I52**	1.37	1.6	1	1.5	240	ADEQUATE WEIGHT	ADEQUATE	13.06	12.29	35
2021	Infante	**Infant 6**	**I38**	2.66	2.6	2.6	3.5	240	ADEQUATE WEIGHT	ADEQUATE	36.04	16.77	39

Section 3: Parameters used to estimate obesity, overweight and hypertension

The classification of the variable "BMI diagnosis" was obtained by calculating the body mass index (BMI) in the following way:

$$BMI = \frac{Weight\ (kg)}{Size\ mts^2}$$

The BMI of each individual in the study was categorized according to the BMI cut-off points where: BMI between 18.5-24.9 is considered normal weight, BMI between 25-29.9 is considered overweight and BMI >= 30 is considered obese. On the other hand, the classification of the variable "fat percentage diagnosis" of the individuals was performed by means of bioimpedance measurement in which "obesity" was determined for people with high and very high fat percentage according to the cut-off points in (table #):

SEXO	Age	Low	Normal	Hight	Very Hight
Woman	20-39	<21	21-32,9	33-38,9	>= 39
	40-59	<23	23-33,9	34-39,9	>=40
	60-79	<24	24-35,99	36-41,9	>=42
Man	20-39	<8	8-19.9	20-24,9	>=25
	40-59	<11	11-21,9	22-27,9	>=28
	60-79	<13	13,24-9	25-29,9	>=30

The cardiovascular risk variable was considered using the blood pressure classification described by the American Heart Association (AHA) based on the category of high blood pressure with a systolic pressure >=130 mmHg and diastolic pressure >= 80 mmHg.
